# Supplementary material for: Mesoscale functional architecture in medial posterior parietal cortex
Source: eLife. 2026 Sep 2;14:RP105213. doi: 10.7554/eLife.105213 (PMC13537763; doi:10.7554/eLife.105213)
Supplement: Peer review history

# Editor report: eLife Assessment

**DOI:** 10.7554/eLife.105213.3.sa0

**Authors:** Leopoldo Petreanu

**Affiliations:** Champalimaud Center for the Unknown Portugal

**Keywords:** Compelling, Fundamental

This **fundamental** study measures the functional specialization of distinct subregions within the mouse posterior parietal cortex (PPC) using mesoscopic two-photon calcium imaging during visual discrimination and choice history-dependent tasks. It presents **compelling** evidence supporting the existence of functional specialized subregions within the PPC. The work will be of interest to system and computational neuroscientists interested in decision-making, working memory, and multisensory integration.

# Referee report: Reviewer #1 (Public review)

**DOI:** 10.7554/eLife.105213.3.sa1

Summary:

This study examined the functional organization of the mouse posterior parietal cortex (PPC) using meso-scale two-photon calcium imaging during visually-guided and history-guided tasks. The researchers found distinct functional modules within the medial PPC: area A, which integrates somatosensory and choice information, and area AM, which integrates visual and choice information. Area A also showed a robust representation of choice history and posture. The study further revealed distinct patterns of inter-area correlations for A and AM, suggesting different roles in cortical communication. These findings shed light on the functional architecture of the mouse PPC and its involvement in various sensorimotor and cognitive functions.

Strengths:

Overall, I find this manuscript excellent. It is very clearly written and built up logically. The subject is important, and the data supports the conclusions without overstating implications. Where the manuscript shines the most is the exceptionally thorough analysis of the data. The authors set a high bar for identifying the boundaries of the PPC subareas, where they combine both somatosensory and visual intrinsic imaging. There are many things to compliment the authors on, but one thing that should be applauded in particular is the analysis of the body movements of the mice in the tube. Anyone working with head-fixed mice knows that mice don't sit still but that almost invariable remains unanalyzed. Here the authors show that this indeed explained some of the variance in the data.

Comments on revisions:

I only had minor comments on the first version of the manuscript and these concerns were fully addressed after revision.

# Referee report: Reviewer #2 (Public review)

**DOI:** 10.7554/eLife.105213.3.sa2

Summary:

The posterior parietal cortex (PPC) has been identified as an integrator of multiple sensory streams and guides decision making. Hira et al observe that dissection of the functional specialization of PPC subregions requires simultaneous measurement of neuronal activity throughout these areas. To this end, they use widefield calcium imaging to capture the activity of thousands of neurons across the PPC and surrounding areas. They begin by delineating the boundaries between the primary sensory and higher visual areas using intrinsic imaging and validate their mapping using calcium imaging. They then conduct imaging during a visually guided task to identify neurons that respond selectively to visual stimuli or choice. They find that vision and choice neurons intermingle primarily in the anterior medial (AM) area, and that AM uniquely encodes information regarding both the visual stimulus and the previous choice, positioning AM as the main site of integration of behavioral and visual information for this task.

Strengths:

There is an enormous amount of data and results reveal very interesting relationships between stimulus and choice coding across areas and how network dynamics relate to task coding.

Weaknesses:

The enormity of the data and the complexity of the analysis makes the manuscript hard to follow. Sometimes it reads like a laundry list of results as opposed a cohesive story.

Comments on revisions:

The authors have addressed our concerns.

# Referee report: Reviewer #3 (Public review)

**DOI:** 10.7554/eLife.105213.3.sa3

Summary:

This work from Hira et al leverages mesoscopic 2-photon imaging to study large neural populations in different higher visual areas, in particular areas A and AM of the parietal cortex. The focus of the study is to obtain a better understanding of the representation of different task-related parameters, such as choice formation and short-term history, as well as visual responses in large neural populations across different cortical regions to obtain a better understanding of the functional specialization of neural populations in each region as well as the interaction of neural populations across regions. The authors image a large number of neurons in animals that either perform a visual discrimination or a history-dependent task to test how task demands affect neural responses and population dynamics. Furthermore, by including a behavioral perturbation of animal posture they aim to dissociate the neural representation of history signals from body posture. Lastly, they relate their functional findings to anatomical data from the Allen connectivity atlas and show a strong relation of functional correlations on anatomical connectivity patterns.

Strengths:

Overall, the study is very well done and tackles a problem that should be of high interest to the field by aiming to obtain a better understanding of the function and spatial structure of different regions in the parietal cortex. The experimental approach and analyses are sound and of high quality and the main conclusions are well supported by the results. Aside from the detailed analyses, a particular strength is the additional experimental perturbation of posture to isolate history-related activity which supports the conclusion that both posture and history signals are represented in different neurons within the same region.

Weaknesses:

The work does not focus on functional overlap at the single-cell level but on the spatial distribution of functional classes across areas. A minor weakness is therefore that it does not explicitly address how the finding of functional clusters relate to established notions of mixed selectivity within PPC.

# Author comment: Author response

**DOI:** 10.7554/eLife.105213.3.sa4

**Authors:** Riichiro Hira, Leah B Townsend, Ikuko T Smith, Che-Hang Yu, Jeffery N Stirman, Yiyi Yu, Spencer LaVere Smith

**Affiliations:** Department of Electrical and Computer Engineering, University of California Santa Barbara, Tokyo Tokyo Japan; Neuroscience Center, University of North Carolina Chapel Hill. Chapel Hill United States; University of California, Santa Barbara Santa Barbara United States; Department of Electrical and Computer Engineering, University of California Santa Barbara Santa Barbara United States; University of California, Santa Barbara Santa Barbara United States; University of California, Santa Barbara Santa Barbara United States; University of California, Santa Barbara Santa Barbara United States

The following is the authors’ response to the original reviews.

We sincerely appreciate your constructive feedback. Based on the comments from the three reviewers, we were able to substantially improve the manuscript. Below, we provide our point-by-point responses.

**Public Reviews:**

**Reviewer #1 (Public review):**

Summary:

This study examined the functional organization of the mouse posterior parietal cortex (PPC) using meso-scale two-photon calcium imaging during visually-guided and history-guided tasks. The researchers found distinct functional modules within the medial PPC: area A, which integrates somatosensory and choice information, and area AM, which integrates visual and choice information. Area A also showed a robust representation of choice history and posture. The study further revealed distinct patterns of inter-area correlations for A and AM, suggesting different roles in cortical communication. These findings shed light on the functional architecture of the mouse PPC and its involvement in various sensorimotor and cognitive functions.

Strengths:

Overall, I find this manuscript excellent. It is very clearly written and built up logically. The subject is important, and the data supports the conclusions without overstating implications. Where the manuscript shines the most is the exceptionally thorough analysis of the data. The authors set a high bar for identifying the boundaries of the PPC subareas, where they combine both somatosensory and visual intrinsic imaging. There are many things to compliment the authors on, but one thing that should be applauded in particular is the analysis of the body movements of the mice in the tube. Anyone working with head-fixed mice knows that mice don't sit still but that almost invariable remains unanalyzed. Here the authors show that this indeed explained some of the variance in the data.

Weaknesses:

I see no major weaknesses and I only have minor comments.

**Reviewer #2 (Public review):**

Summary:

The posterior parietal cortex (PPC) has been identified as an integrator of multiple sensory streams and guides decision-making. Hira et al observe that dissection of the functional specialization of PPC subregions requires simultaneous measurement of neuronal activity throughout these areas. To this end, they use wide-field calcium imaging to capture the activity of thousands of neurons across the PPC and surrounding areas. They begin by delineating the boundaries between the primary sensory and higher visual areas using intrinsic imaging and validate their mapping using calcium imaging. They then conduct imaging during a visually guided task to identify neurons that respond selectively to visual stimuli or choices. They find that vision and choice neurons intermingle primarily in the anterior medial (AM) area, and that AM uniquely encodes information regarding both the visual stimulus and the previous choice, positioning AM as the main site of integration of behavioral and visual information for this task.

Strengths:

There is an enormous amount of data and results reveal very interesting relationships between stimulus and choice coding across areas and how network dynamics relate to task coding.

Weaknesses:

The enormity of the data and the complexity of the analysis make the manuscript hard to follow. Sometimes it reads like a laundry list of results as opposed to a cohesive story.

**Reviewer #3 (Public review):**

Summary: This work from Hira et al leverages mesoscopic 2-photon imaging to study large neural populations in different higher visual areas, in particular areas A and AM of the parietal cortex. The focus of the study is to obtain a better understanding of the representation of different task-related parameters, such as choice formation and short-term history, as well as visual responses in large neural populations across different cortical regions to obtain a better understanding of the functional specialization of neural populations in each region as well as the interaction of neural populations across regions. The authors image a large number of neurons in animals that either perform visual discrimination or a history-dependent task to test how task demands affect neural responses and population dynamics. Furthermore, by including a behavioral perturbation of animal posture they aim to dissociate the neural representation of history signals from body posture. Lastly, they relate their functional findings to anatomical data from the Allen connectivity atlas and show a strong relation between functional correlations on anatomical connectivity patterns.

Strengths:

Overall, the study is very well done and tackles a problem that should be of high interest to the field by aiming to obtain a better understanding of the function and spatial structure of different regions in the parietal cortex. The experimental approach and analyses are sound and of high quality and the main conclusions are well supported by the results. Aside from the detailed analyses, a particular strength is the additional experimental perturbation of posture to isolate history-related activity which supports the conclusion that both posture and history signals are represented in different neurons within the same region. Weaknesses: The main point that I found hard to understand was the fairly strong language on functional clusters of neurons while also stating that neurons encoded combinations of different types of information and leveraging the encoding model to dissociate these contributions. Do the authors find mixed selectivity or rather functional segregation of neural tuning in their data? More details on this and some other points are below.

We thank the three reviewers for their accurate and expert evaluations.

**Recommendations for the authors:**

**Reviewer #1 (Recommendations for the authors):**

(1) It wasn't clear to me why the authors focused on areas A and AM, but not RL. After all, at the beginning of the results, the authors ask: "PPC has been reported to have functions including visually guided decision-making and working memory. Do these functions differ among RL, A, and AM?".

Thank you for the comment. The manuscript first characterizes AM as a region involved in visually guided decision-making and A as a region related to history and/or working memory. Subsequently, when discussing correlation structure, we stated the following:

“In particular, based on the critical functional differences between A and AM that we found, A and AM may belong to distinct cortical networks that consist of different sets of densely interacting cortical areas.”

Thus, the logical flow of our analysis is to first reveal the functional contrast between A and AM through comparative functional analyses across RL, A, and AM, and then to focus on this contrast. We speculate that RL may exhibit more distinctive functional properties in tasks that rely on whisker-based processing or related modalities. We have therefore revised the text as described below to avoid the impression that the manuscript places disproportionate emphasis on RL.

Line 137: “PPC has been reported to have functions including visually guided decisionmaking and working memory. Do these functions differ among A, AM, and RL?”

(2) Figures 2 E, F, and Figure 3A, could the authors indicate the trial structure better on these plots?

Thank you for the comment. We have added explanations of the bar meanings to the figure legends.

Figure 2:

“(E) Representative vision neurons (ROI 1-4 in I). The red bars indicate sampling periods during video presentation, and the brown bars indicate sampling periods without video stimulation. Vertical black lines mark the onset of the sampling period. F. Representative choice neuron (ROI 5-8 in I) and a non-selective neuron (ROI 9). Light blue lines indicate the response periods in trials with left choices, and purple lines indicate the response periods in trials with right choices. Vertical black lines mark the onset of the response period.”

Figure 3:

“(A) The representative history neurons. Numbers correspond to that of panel B and C. Light blue lines indicate rewards delivered from the left lick port, and purple lines indicate rewards delivered from the right lick port. Vertical white lines mark the onset of the sampling period.”

(3) There are several typos that need correcting. Also, small and big capital letters to demark the panel names in the legends have been mixed.

Thank you for the comment. We have corrected the panel labels as described below.

Figure 2 legend:

“Representative choice neuron (ROI 5-8 in I) and a non-selective neuron (ROI 9)”

Figure 3 legend:

“..than the next choice. I. The decoding accuracy of the next choice …”

Figure 3 legend:

“Error bars, mean ± s.e.m. in I, 95% confidence interval in G. M, and O.”

Supplementary Figure 6：

“…neurons with rt ≥ 0.3 (blue) were shown. B. Trial-to-trial activity fluctuation … (rt ≥ 0.3, panel B) was color coded…”

We thoroughly checked the manuscript for typographical errors and corrected the issues.

(4) Many in the field still use the Paxinos nomenclature for PPC subfields, could the authors write something short about how these two nomenclatures correspond?

We have described the relationship between our area definitions and those of Paxinos in the main text as follows.

Line 702: “In addition to our definition, previous studies have also defined posterior parietal cortex (PPC) to include the higher visual areas A, AM, and RL (Glickfeld and Olsen, 2017; Wang et al., 2011). These areas partially overlap with the parietal association regions defined in the Paxinos atlas, including MPtA, LPtA, PtPD, and PtPR. For a detailed discussion of the correspondence and variability among these regional definitions, see Lyamzin and Benucci (2019).”

(5) Analyzing choice history may be affected by the long fluorescence Ca transients and will depend on excellent event deconvolution. Could the authors show some more zoomed-in examples of how well their deconvolution works?

We provide enlarged, trial-by-trial activity traces of the four example neurons shown in Figure 3A in Supplementary Figure 3G. In all neurons, multiple small calcium transients occur repeatedly throughout the delay period, which lasts longer than 10 s. If the sustained activity during the delay were simply due to a long decay time constant, one would expect a large calcium transient in the preceding trial that slowly decays over the delay period. However, such a pattern is not observed in the actual data. Also, since the decay time constant of GCaMP6s is on the order of ~1 s, signals persisting for ~10 s cannot be explained by slow decay alone.

(6) The authors write: "the history neurons exhibited properties of working memory." However, note that this is not a working memory task since the mice don't need to keep evidence in memory, the direction to lick can be made at the very beginning of a trial.

Behaviorally, demonstrating that an animal maintains working memory requires showing that its behavior changes based on retained information when new information is introduced, as in delayed match-to-sample tasks. In the present task, however, the correct action for the next trial is determined at the moment the action in the previous trial is completed, such that animals can simply switch to motor preparation at that point. Thus, from a strictly behavioral perspective, working memory is not required.

On the other hand, during the inter-trial interval (ITI), information from the previous trial dominates over information from the upcoming trial (Fig. 3H), which is more consistent with retention of past information than with motor preparation. Moreover, trials in which neural activity maintained information about the previous trial’s action were associated with a higher probability of correct performance in the subsequent trial. In other words, retaining past information contributes to guiding correct behavior in the next trial.

Based on these neural analyses, we interpret that mice retain information about their previous trial’s action history in working memory and use it to determine behavior in the subsequent trial. Accordingly, we consider ITI activity in PPC to reflect working memory rather than motor preparation. Nevertheless, we acknowledge that your concern is valid, and we have therefore revised the text as follows:

Line 234: “These results suggest that the history neurons exhibited properties of working memory.”

(7) In the section about the Choice History Task, the authors write: "Since the visual stimuli were randomly presented during the sampling period, the mice had to ignore the visual stimuli." Why continue to present the visual stimuli?

Thank you for the suggestion. By designing the vision task and the history task to have identical structures, we can apply the same encoding and decoding models to both tasks, which facilitates direct comparison between them. This design makes it easier to examine how neuronal activity patterns change depending on task demands.

**Reviewer #2 (Recommendations for the authors):**

(1) I don't understand the logic of Figure S7 and the neuropil analysis in general. Neuropil activity is purported to represent input, so it seems unsurprising that nearby neurons would exhibit similar dynamics.

Thank you for your comment. Your argument is correct, and it is not at all surprising that neuropil signals correlate with the activity of surrounding neurons. Here, we quantitatively examined the relationship between neuropil activity and the average activity of nearby neurons. In addition, in a separate analysis, we clarified the relationship between connectome information and neuropil activity. Taken together, these analyses reveal the relationship between connectome information and the local average of neuronal activity. We describe this point as follows:

“Indeed, the trial-to-trial variation of a neuropil activity could be approximated by the average of 1,000–10,000 neurons within several hundred micrometers from the center (Figure S7).”

Although we analyzed this phenomenon in the cases of areas A and AM, this finding should not be considered specific to A and AM but instead has broader, general significance. Accordingly, we added a new Results subsection and revised the manuscript as follows.

Line 448: “Constraints and limits of anatomical connectivity on neuronal population activity Although we have so far focused on the differences between A and AM, our data provide broader insights into the relationship between anatomical connectivity and neuronal population activity. First, based on Figure S7 and the considerations above, anatomical input correlations strongly constrain the correlations between local averages of activity across thousands of neurons. We then asked whether this anatomical constraint extends beyond mean activity, and how anatomical input correlations relate to relationships between neuronal population activities (population vectors).

The correlation between *CC_t_* and *r_anatomy_* was moderate (r = 0.60, Figure 6L). This moderate correlation did not change when the coupling neurons were eliminated (r = 0.61). Interestingly, the largest canonical component was the most unpredictable from the anatomical data (Figure 6M). Thus, while inter-area correlations based on the mean activity of neuronal populations are largely determined by anatomical input correlations, correlations between population vectors contain additional structure that cannot be captured by anatomical input correlations alone.

One possible source of this additional structure is globally shared activity, which may reflect behavior, brain state, or levels of neuromodulators. To evaluate the contribution of global activity on the canonical correlation between areas, we first compared the canonical coefficient vectors (CCV). We found that the first CCV had a similar orientation, regardless of the paired areas (Figure6N). This indicates that the largest components of correlated activity in the CCA analysis are globally shared fluctuations. We also directly evaluated the correlated activity components across all 8 areas with generalized canonical correlation analysis. The first CCV also had a similar orientation to the first generalized canonical coefficient vector (GCCV) (Figure 6O). These results indicate that the largest canonical component reflects a global correlation across all cortical areas imaged. Such global correlations may be driven by factors beyond cortico-cortical or thalamo-cortical inputs, such as the animal’s behavioral state as we recently characterized (H. Imamura et al., 2025; F. Imamura et al., 2025). We also confirmed the robustness of these results by repeating analyses using only the 40% highly active neurons after denoising with non-negative deconvolution (36828 out of 91397 neurons; Figure S9).”

(2) Furthermore, the neuropil signal likely contains signals from out-of-focus neurons that are presumably functioning similarly to the in-focus cells. Wouldn't the interesting question be to what extent the local neuropil signal in, for example, area A resembled that of neuronal activity in S1t?

Thank you very much for your comment. We agree with your point. Based on the evaluation in Figure S7, the neuropil signal likely contains the average activity of several thousand local neurons, including out-of-focus contributions. The neuropil signal in area A may also partially reflect neuronal activity from the neighboring S1t area. In particular, neurons that show little correlation with the local population average (i.e., the neuropil signal) within the same area are sometimes referred to as “soloists” (M. Okun et al., 2015). If such soloist neurons were found to exhibit strong correlations with the neuropil signal of an adjacent area, this would be a highly interesting result. However, such an analysis would go beyond the scope of the present manuscript and would require a new line of discussion; therefore, we plan to address this issue in future work.

(3) I generally found the final Results section (Relationship between mesoscale functional correlation and anatomical connections) to be hard to follow. The motivation for this analysis should be better explained.

We fully incorporated your suggestion and rewrote the final section of the Results accordingly. Please refer to our responses to the two comments above.

(4) The question of brain state/neuromodulation as a driver of the globally shared activity may be addressable by considering its correlation with pupillometry data.

We fully agree with your suggestion. In our experiments, visual stimuli change continuously, and thus pupil diameter changes are most likely driven primarily by changes in visual input. Although state-dependent fluctuations of brain activity may also be present, they are likely masked by the larger effects induced by visual stimulation. Therefore, analyzing pupil-linked signals as a factor of globally shared activity would be more appropriately addressed in experiments without visual stimulation. We plan to investigate this issue in future studies. Here, we have added the following description regarding pupil dynamics and their associated relationships.

Line 292: “We found that the neurons related to the tail and forepaws were similarly distributed around the parietal cortex including S1 and A, while the pupil-size related neurons were mapped around visual areas (Figure 4C). Changes in pupil diameter may influence neuronal activity through multiple mechanisms, including behavioral state or noradrenergic level [REF], nonlinear interactions with visual stimulation, and changes in the amount of light reaching the retina.”

Minor issues

(1) The authors deploy sophisticated mathematical techniques with essentially no explanation outside the Methods section. A brief introduction of jPCA and CCA in the main text would help the reader understand the value of these analyses.

Thank you for the comment. We added the following explanation.

Line 238: “In this task, left and right selection are alternated, so the activity of the history neuron is a sequence that repeats in two consecutive trials. We used jPCA^49^ to visualize and quantify this activity pattern (Figure 3K). jPCA identifies low-dimensional projections of population activity that maximize rotational dynamics across time.”

Line 374: “Next, to investigate *r_t_* of the population activity (*r_t_population_*), we first reduced the dimension of population activity in each area into 10 by using PCA (principal component analysis) (Figure S6B,C). Then, “fluctuation activity” was recalculated for each dimension and trial type, analogous to the single-neuron analysis described above, but here representing noise in population-level activation patterns. We applied CCA (canonical correlation analysis) to each pair of areas and obtained an average of 10 canonical correlations (*CC_t_*) as *r_t_population_*. CCA identifies pairs of linear combinations of population activity from two areas that maximize their correlation across trials, thereby capturing shared population-level fluctuations. The *CC_t_* structure between areas was similar across task types (Figure 5H) indicating that this structure reflects the underlying functional connectivity independent of the task. The *CC_t_* between A and S1t was the largest among all the pairs (Figure 5H), whereas when the *CC_t_* was averaged across all connections for each area, A and AM had the largest and second largest *C_t_*, respectively (Figure 5I). The dominance in *CC_t_* in A and AM disappeared when the neurons with *r_t_single_* >0.3 were removed. Notably, the *CC_t_* of AM and the other areas was uniform regardless of the paired areas across all 10 canonical components (Figure 5J). Thus, area AM is an integration hub of interareal communication, whereas A simply coupled with S1t, and such correlation structure at the population level critically depends on this subset of neurons.”

(2) The manuscript contains numerous typos ("hoice"), spelling errors ("parameters", "costom"), abbreviations that are not defined (ex: RL/rostrolateral), and minor grammatical issues that should be addressed by a round of copy editing.

We thank the reviewer for pointing this out. We have thoroughly corrected these typographical and grammatical errors, and have described the revisions in detail in our response to Reviewer 1, comment (3). In addition, we have clarified the abbreviations in the manuscript as follows.

Line 94: “rostrolateral area (RL)”

Figure 1 legend: “Abbreviations: RL, rostrolateral HVA; PM, posteromedial HVA; RSC, retrosplenial cortex.“

(3) Figure 3K unlabeled axes.

Thank you for the comment. We have added the axis labels.

(4) Figure 3K caption, first "(right)" should be "(left)".

Thank you very much for your careful attention to detail. We have made the requested correction.

(5) Figure 6 is hard to read. Panel A is too small, and the interpretation of G is difficult.

- For panel A, we added an enlarged view with images from a larger number of trials in Figure S7A.

- G represents the connectivity matrix. The sources correspond to the injection sites, and the targets correspond to voxels in the cerebral cortex. Because the latter may not be immediately clear, we explicitly indicated in the figure that the targets are cortical voxels.

(6) Figure S4C has a double compass.

Thank you for the comment. We have revised the manuscript accordingly.

**Reviewer #3 (Recommendations for the authors):**

While I have some questions and additional suggestions to further improve the clarity of the manuscript, I already found it to be highly interesting and well done in its current form.

Major points:

(1) The t-SNE comes up rather abruptly and is not well-explained in the main text or the figure caption. It would be good to provide some more information on the rationale of this analysis and how to interpret it. In particular, I don't see clear clusters in Figure 2H although the description of the authors seems to indicate that they observe clear functional classes such as choice, stimulus, and history neurons. Similarly, in Figure 3B, I don't see a clear separation between history and choice neurons in the t-SNE map. The example cells in Figure 3A appear to be delayed or long-tailed choice neurons rather than a dedicated group of 'history neurons'. It would be helpful for the interpretation of the t-SNE plots to show different PSTHs for different regions of the t-SNE map to better illustrate what different regions within the t-SNE projection represent and what distinguishes these cells.

Thank you for the comment. The absence of clearly defined clusters in the t-SNE map suggests that neuronal activity forms a continuum rather than discrete classes. Importantly, the purpose of the t-SNE map here is not to identify sharp clusters, but to demonstrate that the functional categorization provided by our encoding model broadly and comprehensively spans the major structures present in the unsupervised t-SNE map. We have revised the relevant text in the manuscript accordingly as follows.

Line 158: “To examine whether the neuron groups labeled by this model broadly capture the diversity of neuronal activity, we performed unsupervised clustering of neuronal activity using t-SNE. The functional labels revealed by this encoding model were consistent with the t-SNE clusters, indicating the validity of the encoding model (Figure 2H; Figure S4B; materials and methods).”

The issue regarding History neurons was also raised in Reviewer #1’s comment (5). We provide an enlarged view of Figure 3A in Figure S3A. Each History neuron exhibits multiple calcium transients repeatedly and asynchronously following the previous reward acquisition. Therefore, rather than being “choice neurons with a long tail,” these neurons are better interpreted as neurons whose activity is sustained during this delay period.

(2) Although the authors mention that neurons represent a mixture of features, they then use the encoding model to isolate clusters, such as vision or choice neurons. In general, the language throughout the manuscript suggests that there are various clusters of functionally segregated neurons (vision, choice, history, or coupling neurons). However, it is not clear to me to what extent this is supported by the data. Couldn't a choice neuron also be a vision neuron if both variables make significant contributions to the model? Similarly, are 'history' and 'choice' separate labels from the encoding model, or could a cell be given multiple labels? If a cell could be given multiple labels how did the authors create the colored plots on the right-hand side of Figures 2H and 3B? The example history cells in Figure 3J also appear to be highly selective for the contralateral choice, so again this seems to argue against a clear separation of choice and history neurons.

Each label is assigned based on whether the corresponding coefficient is significant in the encoding model, and therefore neurons that are both vision- and choice-selective do exist. The presence of mixed selectivity neurons in PPC is well established (e.g., MJ Goard et al., 2016 elife). In this manuscript, however, we focus not on functional overlap at the single neuron level, but on the spatial distribution of functional classes, and thus do not explicitly address mixed selectivity. Although the colors in Figure 2H and Figure 3B overlap, the underlying data for each are presented separately in Figure S4B and S4D, respectively. As shown there, each color generally occupies distinct regions in the t-SNE map.

(3) The decoding analysis in Figure 3F also suggests that a potential reason why there are more choice history signals in areas S1 and A is that neural activity is simply larger rather than due to the activity of a dedicated group of history neurons. Are the authors interpreting this differently? Could the duration of stored choice information also be affected by the dynamics of the calcium indicator?

Thank you for the comment. Simply having larger neural activity in S1t or A would not result in calcium transients with a ~1-s time constant persisting throughout a delay period lasting up to 10 seconds. As also noted in comment (1), History neurons exhibit sustained and repeated calcium transients, and therefore their activity cannot be explained merely by elevated neural activity levels. One could argue that all cortical areas carry history-related information but that the signal-to-noise ratio is higher in S1t or A, which might make such signals more detectable there. If this were the case, however, differences across areas in all forms of selectivity should similarly depend on signal-to-noise ratio. This is not what we observe in our data.

(4) I'm confused as to why the decoding accuracy is so high for areas A and S1t at time -3 relative to the choice in Figure 3F. Shouldn't this be the same as predicting the next choice in Figure 3H? Why is the decoding accuracy lower in this case?

Thank you for the comment. The analysis shown in Figure 3F includes only trials in which the choice was correct. This is the reason why the decoding performance in Figure 3H is lower. We have added this clarification to the main text.

Figure 3F: “Decoding accuracy of choice, outcome, and visual stimuli by the activity of 20 neurons from each area using only correct trials, before and after the choice onset, reward delivery, and the end of the visual stimuli, respectively. Line colors corresponded to the areas shown in panel G.”

(5) In general, the text is not very detailed about the statistics. While test scores and p-values are mentioned, it would be good to also state what is actually compared and what the n is (e.g. how many neurons, neuron pairs, areas, sessions, or animals) for each case. How do the authors account for the nested experiment design where many neurons are coming from a low number of animals?

Thank you for the comment. In our decoding analyses, we generally treat the number of animals as the independent variable. In contrast, for the encoding model analyses, we treat the number of neurons as the independent variable. As you correctly pointed out, because we recorded activity from a large number of neurons, statistical tests that treat individual neurons as independent samples can readily yield significant p-values even with a small number of animals. We have therefore confirmed that our conclusions are not driven by a large effect from a single animal. When making qualitative claims, we rely not only on statistical significance (p-values) but also require clear differences in effect size. We have added the following clarification to the Statistics section accordingly.

Line 1049: ”For the decoding analyses, the number of animals was treated as the independent variable, whereas for the encoding model analyses, the number of neurons was treated as the independent variable. To ensure that the results were not driven by a single animal, we repeated the statistical tests while systematically excluding data from one animal at a time and confirmed that statistical significance was preserved in all cases. Furthermore, qualitative interpretations were made only when differences in effect size were clearly observed.”

(6) How was the grouping in Figure 2O done? Specifically, how were the thresholds for the dashed lines selected to separate PM and V1 from AM and RL as association areas? It seems to me like this grouping was done rather arbitrarily as the difference in choice decoding accuracy is not particularly large between these areas.

This line does not have a specific quantitative basis, but we consider it useful as an illustrative aid. We have added this clarification to the figure legend.

Figure 2O: “Decoding accuracies of time in video presentation and choice direction indicate that AM would be the best position for associating these two signals. The background color and dashed lines are provided as visual aids for illustrative purposes.”

(7) The fact that neurons with high rt_single tend to share the same function might also indicate the approach is insufficient to remove all effects of tuning to trial types from the neural data. Since the authors subtract the average of each trial type, the average trial-type related information is removed but type-specific variations that are not equally presented in the average might remain. For choice neurons for example, attentive vs in-attentive choices could be represented differently and thus remain in the data since the average would be a mixture of both. The same goes for other factors that would drive a particular modulation in the choice - or stimulus - related part of the trial which could still tie these neurons together. One way to circumvent this concern could be to first compute the mean activity for all time points in each trial and then compute the trial-to-trial variability across all trials of the same type. Alternatively, I would be curious how the results play out when using data when the animal is not actively performing the task to compute rt_single.

Thank you for the comment. The concern raised by the reviewer applies to all noise-correlation analyses and highlights an important limitation of this approach, namely that factors other than the observed variables are treated as noise. By subtracting the trial-averaged activity, information related to sensory input and the direction of the first lick at choice can be removed. However, other factors cannot be eliminated if they are not observed. For example, if right hindlimb movements tend to occur only in trials with visual stimulation combined with left choice, such effects cannot be removed because they are not measured. The same issue remains even when restricting the analysis to a single trial type. Based on these considerations, we have added the following text to the manuscript.

Line 932: “Correlation of trial-to-trial variance of activity between a pair of single neurons was defined as *r_t_single_*. To calculate *r_t_single_*, we averaged the activity of individual neurons over the sampling period, and the average across each trial type was subtracted from this value. The trial types consisted of four sets of pairs of stimuli and responses, that is, the video stimulation and left choice, the video stimulation and right choice, the black screen and left choice, and the black screen and right choice. By this operation, we extracted the fluctuating components of single-neuron activity that are independent of the trial types. Although the finding that neurons with high *r_t_single_* tend to share the functional properties we propose is not a trivial consequence of the analysis. At the same time, it remains possible that high *r_t_single_* reflects the degree to which neurons share unobserved features, and that such features are correlated with our functional classification. Thus, while this analysis suggests that correlated fluctuations across cortical areas may contribute to the determination of functional types, establishing an exclusive conclusion will require more fine-grained behavioral measurements, tighter control of internal states, and causal identification through targeted interventions.”

Minor points:

(1) Why did the authors use the activity of 50 neurons for the decoder analysis in Figure 2K? Didn't they have many more neurons available? How were these selected?

We found that the conclusions were identical when using datasets consisting of either 50 neurons or 20 neurons across all analyses. Because the total number of recorded PM neurons did not reach 100 in at least one mouse, we standardized the analyses to 50 neurons in order to match the number of neurons across all cortical areas and animals.

(2) The authors mention that some PPC neurons showed complex dynamics rather than encoding a specific feature such as visual or choice information but do not mention actual numbers on this point. It would be good to quantify to what extent neurons in different regions represent such mixed selectivity and whether there are clear differences in selectivity. This would also be interesting to discuss in context to earlier work on mixed selectivity in the parietal cortex, such as Raposo et al 2015.

Thank you for the comment. Your point is entirely valid. However, as explained in our response to your major comment, our analyses focus not on how individual neurons are classified, but rather on the spatial distribution of these functional categories.

(3) I have a hard time understanding what the length of the bars in the right panel of Figure 2k indicates. Does this plot show more than the decoder accuracy before and after the choice? Is the bar length related to the standard deviation? The same question for the visualization in panel 2n. It looks nice but I'm confused about what it shows exactly.

These bars represent confidence intervals. Although this is stated at the end of the Figure 2 legend, we agree that it may not be sufficiently clear, and we have therefore added this information to the Statistics section.

Line 1046: “In Figure 2K and N, and Figure 3G, L, M, and O, the bars indicate the 95% confidence intervals. All other bars denote s.e.m., unless otherwise noted.”

(4) Is Figure 3D showing the same association index as in Figure 2j, thus showing the same result as in the vision task or is this meant to show something new? It was not clear to me from the wording, so it would be good to clarify.

You are correct that the magenta trace in Fig. 3D is the same as in Fig. 2J. This panel was included to explicitly illustrate that, in areas A and AM, the separation between History and Association approximately overlaps. We have added the following clarification to the figure legend accordingly.

Figure 3D: “The percentage of history neurons and the association index (as defined in Fig. 2J) were overlaid for comparison.”

(5) When computing the Pseudo R2 for regressor contribution, how was the null model computed? From shuffling all regressors in the model? I think this is fine but it's not fully clear what the intended effect of this procedure is. For the description of Figure 4C it would be good to add a sentence explaining how to interpret the pseudo R^2.

The null model predicts a fixed value that is independent of the explanatory variables, i.e., it predicts only the intercept. This provides a useful correction term when performing cross-validation, particularly in cases where baseline values differ across folds. In Figure 4C, the analysis shows the contribution of adding body part positions and pupil diameter to the model for predicting neural activity. We have added the following text to the Methods section.

Line 881: “To estimate the contribution of parameters for the left forelimb, the right forelimb, the tail, and the pupil, we repeated the same analysis with a reduced model where each set of predictors was eliminated from the full model (Figure 4B). Then, the pseudo-R^2^ was obtained for each set of predictors by (*MSE_reduced_* ‒ *MSE_full_*) /*MSE_null_*, where MSE is the mean squared error, *MSE_reduced_* is MSE for the reduced model, *MSE_full_* is the MSE of the full model, and *MSE_null_* is the null model. The null model predicts a fixed value that is independent of the explanatory variables; specifically, it simply outputs the mean of the training data. For example, we constructed a regression model without the parameters regarding the left forelimb (green shade of Figure 4B), obtained *MSE_reduced_* for the left forelimb, and the pseudo-R^2^ was calculated as above by comparing the MSE of the full model and the null model. This value reflects the extent to which the position of the left forelimb contributes to the prediction of neuronal activity.”

(6) It seems surprising that the pupil-size-related neurons were mapped around visual areas although the pupil should carry clear luminance information. Is this because the luminancerelated information in the pupil can also be explained by the stimulus variable in the model?

Pupil size changed markedly before and after visual stimulus presentation (Figure S5C), dilating during the black stimulus and constricting during the video stimulus. This likely reflects changes relative to the luminance of the gray screen presented in the absence of visual stimuli. In our encoding model, visual stimuli are included as independent regressors for each corresponding time window. Therefore, pupil fluctuations that are temporally locked to visual stimulation are explained by these visual regressors. Neuronal activity that is better explained by pupil size changes not accounted for by the visual regressors is classified as pupil-related. At least three mechanisms may underlie the influence of pupil size on neuronal activity. First, fluctuations in pupil diameter have been linked to behavioral state or noradrenergic level [REF], which can act as variables independent of visual stimulation. Second, pupil fluctuations may be amplified in a stimulus-dependent manner, reflecting nonlinear interactions between visual input and brain state. Third, changes in pupil diameter alter the amount of light reaching the retina, which can modulate activity in visual cortical areas. The latter two mechanisms are therefore expected to predominantly affect visual areas and may explain why pupil-related neurons are more frequently observed there. The first mechanism is likely related to global brain state, and its association with behavior may account for the presence of pupil-related neurons in S1. However, these interpretations require confirmation through more refined causal manipulations. Accordingly, we limited the addition to the manuscript to the following statement.

Line 292: “We found that the neurons related to the tail and forepaws were similarly distributed around the parietal cortex including S1 and A, while the pupil-size related neurons were mapped around visual areas (Figure 4C). Changes in pupil diameter may influence neuronal activity through multiple mechanisms, including behavioral state or noradrenergic level [REF], nonlinear interactions with visual stimulation, and changes in the amount of light reaching the retina.”

(7) What is meant by 'external control parameters such as a video frame' when explaining the encoding model?

Thank you for the comment. We added the following explanation.

Line 151: “In the encoding model, the activity of each neuron was fitted by a weighted sum of external control parameters, such as video frames, and behavioral parameters, such as choice and reward direction. Because the visual stimulus changes continuously over time, sliding time windows were placed during the visual stimulus period.”

(8) What does the trace in Figure 2G show? Is this a single-cell example? What are the axes here?

We added an explanation to the figure legend.

Figure 2G: “Schematic of our encoding model. The bottom right panel shows an example of single-neuron activity with an overlay of the fitting obtained by the encoding model.”

(9) There seems to be a word missing in the sentence that describes the results for Figure 3O in the main text.

Thank you for the comment. We added the following description related to Fig. 3O.

Line 247: “resulting in the decoding accuracy of time after a specific choice being lower than in A (Figure 3O).”

(10) The abbreviation RP is used when describing Figure S5A. It should be mentioned that this refers to the response period.

Thank you for the comment. We added the following description related to Figure S5A.

Line 283: “We found that the angle of the tail was significantly different from the baseline values several seconds after the response period (RP) (Figure S5A)”

(11) I can't see the color difference between the traces in Figure 2E. There are probably red and green but this is hard to see for readers with red-green color blindness. Does the black indicate the time of visual stimulation? Is the line in Figure 2F the time when the spouts move in?

Thank you for the comment. In Fig. 2E, we improved visibility by changing the line opacity. In addition, the vertical line in Fig. 2E indicates the onset of the visual stimulus, and the vertical line in Fig. 2F indicates the onset of the response period. We have added the following explanations to the figure legend.

Figure 2: E. “Representative vision neurons (ROI 1-4 in I). The red bars indicate sampling periods during video presentation, and the brown bars indicate sampling periods without video stimulation. Vertical black lines mark the onset of the sampling period. F. Representative choice neuron (ROI 5-8 in I) and a non-selective neuron (ROI 9). Light blue lines indicate the response periods in trials with left choices, and purple lines indicate the response periods in trials with right choices. Vertical black lines mark the onset of the response period.”

(12) It might be useful to provide a short explanation in the results or methods of why the harmonic mean was used for the computation of the association index. I think it makes sense but since it is not commonly used this could be helpful for the reader to understand the approach.

Thank you for the comment. We added the following explanation to the main text.

Line 869: “The association index was determined by the harmonic mean of the rates of vision neurons and choice neurons. The harmonic mean approaches the arithmetic mean when the two values are similar, but becomes closer to the smaller value when the two values differ substantially. Therefore, the association index takes a large value when both vision neurons and choice neurons are abundant.”

(13) I don't fully understand how coupling diversity is computed. If there are six preference vectors, what is meant by taking the average of angles between all pairs of the two vectors?

Which two are meant here?

Thank you for the comment. We revised the explanation as follows.

Line 950: “To quantify the diversity of coupling patterns across clusters, we computed the angle between every pair of preference vectors. We then averaged these pairwise angles and defined this quantity as the “coupling diversity.”

(14) The results text states that the high correlation between r_anatomy and r_neuropil (Figure 6I) is evidence for the functional correlations being driven by cortico-cortical connectivity. However, Figure 6J shows that correlations for either cortico-cortical or thalamo-cortical connectivity are below 0.94 and generally higher for thalamo-cortical connectivity. This doesn't negate the general point of the authors but it would be good to clarify this section so it is easier to understand if r_anatomy includes both cortico-cortical and thalamo-cortical data and how the results in Figure I and J go together with the description in the results section.

You are correct. We have revised the text to clarify that the analysis reflects the combined effects of both cortico-cortical and thalamo-cortical inputs.

Line 436: “This correspondence suggests that the mesoscale interarea correlation is determined by the cortico-cortical and thalamo-cortical common input at mesoscale. Figure S8: A. Using Allen connectivity atlas, the axonal density of cortico-cortical and thalamo-cortical projection was analyzed.”

(15) I'm not very familiar with canonical correlation analysis and found this part hard to follow. Some additional explainer sentences would be helpful here. For example, what does it mean to take the average of the top 10 canonical correlations as rt_population? What exactly are the canonical correlation vectors? It was also not clear to me what exactly the results in Figure 5J signify.

Thank you for the comment. We have clarified the description in the main text related to CCA and the associated analyses as follows.

Line 374: “Next, to investigate *r_t_* of the population activity (*r_t_population_*), we first reduced the dimension of population activity in each area into 10 by using PCA (principal component analysis) (Figure S6B,C). Then, “fluctuation activity” was recalculated for each dimension and trial type, analogous to the single-neuron analysis described above, but here representing noise in population-level activation patterns. We applied CCA (canonical correlation analysis) to each pair of areas and obtained an average of 10 canonical correlations (*CC_t_*) as *r_t_population_*. CCA identifies pairs of linear combinations of population activity from two areas that maximize their correlation across trials, thereby capturing shared population-level fluctuations. The *CC_t_* structure between areas was similar across task types (Figure 5H) indicating that this structure reflects the underlying functional connectivity independent of the task. The *CC_t_* between A and S1t was the largest among all the pairs (Figure 5H), whereas when the *CC_t_* was averaged across all connections for each area, A and AM had the largest and second largest *CC_t_*, respectively (Figure 5I). The dominance in *CC_t_* in A and AM disappeared when the neurons with *r_t,single_* >0.3 were removed. Notably, the *CC_t_* of AM and the other areas was uniform regardless of the paired areas across all 10 canonical components (Figure 5J). Thus, area AM is an integration hub of interareal communication, whereas A simply coupled with S1t, and such a correlation structure at the population level critically depends on this subset of neurons.”
